# Learning from COVID-19 related trial adaptations to inform efficient trial design—a sequential mixed methods study

**DOI:** 10.1186/s12874-022-01609-6

**Published:** 2022-04-29

**Authors:** Robin Chatters, Cindy L. Cooper, Alicia O’Cathain, Caroline Murphy, Athene Lane, Katie Sutherland, Christopher Burton, Angela Cape, Louis Tunnicliffe

**Affiliations:** 1 Sheffield Clinical Trials Research Unit, School of Health and Related Research, The University of Sheffield, Regents Court, Regent Street, Sheffield, S1 4DA England; 2 Medical Care Research Unit, School of Health and Related Research, The University of Sheffield, Sheffield, UK; 3 King’s Clinical Trials Unit, King’s College London, London, UK; 4 Bristol Randomised Trials Collaboration in the Bristol Trials Centre, Bristol Medical School, University of Bristol, Bristol, England; 5 Academic Unit of Primary Medical Care, The University of Sheffield, Sheffield, UK; 6 London School of Hygiene & Tropical Medicine, London, UK

**Keywords:** Clinical trials, COVID-19, Efficient trial design, Trial methodology, Recruitment, Consent, Follow-up, Intervention delivery

## Abstract

**Background:**

Many clinical trial procedures were often undertaken in-person prior to the COVID-19 pandemic, which has resulted in adaptations to these procedures to enable trials to continue. The aim of this study was to understand whether the adaptations made to clinical trials by UK Clinical Trials Units (CTUs) during the pandemic have the potential to improve the efficiency of trials post-pandemic.

**Methods:**

This was a mixed methods study, initially involving an online survey administered to all registered UK CTUs to identify studies that had made adaptations due to the pandemic. Representatives from selected studies were qualitatively interviewed to explore the adaptations made and their potential to improve the efficiency of future trials. A literature review was undertaken to locate published evidence concerning the investigated adaptations. The findings from the interviews were reviewed by a group of CTU and patient representatives within a workshop, where discussions focused on the potential of the adaptations to improve the efficiency of future trials.

**Results:**

Forty studies were identified by the survey. Fourteen studies were selected and fifteen CTU staff were interviewed about the adaptations. The workshop included 15 CTU and 3 patient representatives. Adaptations were not seen as leading to direct efficiency savings for CTUs. However, three adaptations may have the potential to directly improve efficiencies for trial sites and participants beyond the pandemic: a split remote-first eligibility assessment, recruitment outside the NHS via a charity, and remote consent. There was a lack of published evidence to support the former two adaptations, however, remote consent is widely supported in the literature. Other identified adaptations may benefit by improving flexibility for the participant. Barriers to using these adaptations include the impact on scientific validity, limitations in the role of the CTU, and participant’s access to technology.

**Conclusions:**

Three adaptations (a split remote-first eligibility assessment, recruitment outside the NHS via a charity, and remote consent) have the potential to improve clinical trials but only one (remote consent) is supported by evidence. These adaptations could be tested in future co-ordinated ‘studies within a trial’ (SWAT).

**Supplementary Information:**

The online version contains supplementary material available at 10.1186/s12874-022-01609-6.

## Introduction

Most Clinical Trials Units (CTUs) in the UK are registered with the UKCRC (UK Clinical Research Collaboration) and are responsible for assisting in the grant application process and coordinating the trial if funded [1]. In 2020 a global pandemic (COVID-19) disrupted clinical care and the delivery of research worldwide. In the UK, many clinical trials are embedded within the National Health Service (NHS) and were suspended due to a lack of NHS site research staff, reductions in face-to face contact, and to allow pandemic related studies to take precedence [2, 3]. To restart (or continue) during the pandemic, CTUs, along with clinical investigators in the NHS, had to quickly adapt clinical trial processes, predominately by reducing the need for in-person contact [3]. Some of the main concerns for CTUs were around maintaining recruitment of trial participants, intervention delivery, and data collection, all of which have the potential to be affected by social distancing rules due to the pandemic [3].

The pandemic led to CTU personnel considering remote delivery, for which there is little evidence to suggest how to best design and conduct trial procedures [4]. During the pandemic however, some recommendations regarding how to adapt trials were made, which included the use of electronic consent [5–7], undertaking in-person visits away from main hospitals [8], virtual safety monitoring [6, 9–11] and delivering investigational medicinal products (IMPs) directly to the patient’s home [9, 10, 12]. These trial adaptations may have been useful during the pandemic as an ‘emergency’ measure to enable clinical trials to continue. However, it is unknown whether they may benefit post-pandemic trials, in particular through reducing costs by increased efficiency and retention.

Other studies have assessed the adaptations made to trials during the pandemic [13–15]. A survey of 34 UK based clinical trials of investigational medicinal products (CTIMPs) found that around half of the 16 trials sampled (47%) temporarily halted recruitment procedures during the pandemic, with only four (12%) continuing with modifications to recruitment, CTIMP delivery (including couriering of the IMP, titration or infusion, 18%, *n* = 6) and follow-up processes (including telephone or video conferencing, 53%, *n* = 17) [13]. In another survey of 32 US-based cancer trials, remote data collection was described by the majority of respondents (90%, *n* = 29) as having the potential to improve the conduct of clinical trials [16]. However, these publications are lacking an in-depth assessment of the barriers to implementation, and whether such adaptations would lead to efficiency gains post-pandemic.

The aim of this study was to assess the adaptations that CTUs had made to clinical trials during the COVID-19 pandemic to enable them to continue, and to identify those adaptations that may improve the efficiency of clinical trials after the pandemic. The focus was on three main areas of interest – recruitment, delivery of the intervention and outcome assessment.

## Methods

### Design

This study comprised of four components: a survey of UK CTUs to identify adaptations, a qualitative interview study of CTU personnel who delivered selected adaptations identified in the survey, a literature review to identify previously published evidence regarding the selected adaptations, and a workshop with CTU personnel and patient representatives to review the findings.

### Survey of CTUs

The aim of the survey was to identify studies, which were managed or supported by UK CTUs, that had made adaptations in order to continue the clinical trial during the pandemic.

#### Survey development, data collection and analysis

The questionnaire was developed by RC and pilot tested by selected study collaborators for face and content validity. All Directors of CTUs (*n* = 53) in the UK were sent the online questionnaire in December 2020 by the UK Clinical Research Collaboration (UKCRC), with a reminder one month later. Consent to complete the questionnaire was implied by returning a completed questionnaire. Respondents identified up to four adaptations from their CTU based on the following criteria:

- A randomised trial with major involvement from the CTU;
- An adaptation made to recruitment, intervention delivery, or follow-up procedures in order for the trial to adapt to the impact of COVID-19;
- In the opinion of the individual completing the survey, the adaptation was transferable to other trials and had the potential to improve the efficiency of trials post-pandemic.

Analysis of the survey was descriptive only. The respondent’s description of the adaptation(s) they had made were categorised. Other free text fields were summarised and categorised accordingly.

### Qualitative interviews with CTU personnel

The aim of the qualitative study was to collect in-depth information about selected adaptations by undertaking semi-structured interviews with CTU representatives who were involved in implementing the adaptation (i.e., trial managers), to understand how the adaptation was undertaken, the challenges and benefits of doing so, and the impact on trial efficiency. The study used a phenomenological framework, as interviewees had direct experience of the phenomenon under study.

#### Selection of studies and their associated adaptations

Our research team selected studies and their associated adaptations purposefully, with adaptations selected that were perceived to be applicable to trials across the CTU’s portfolio and were thought to have the potential to improve efficiency (i.e., time and/or cost).

Priority was given to interviewing CTU personnel who had made adaptations to all three of the main areas of interest (recruitment, follow-up, and intervention delivery). Further maximum variation sampling was undertaken to include variation in: the CTU; intervention type (drug/behavioural/physical/surgical); disease area; population age eligibility criteria; target sample size and treatment focus (treatment/preventative).

The studies were initially selected by RC, with the final selection being approved by the project steering group, which comprised of the main study team, plus five UK CTU representatives.

#### Semi-structured interviews

##### Recruitment

Interviewees were identified from the survey responses. Where one or more individual was named as a potential contact on the survey, the individual thought to be most involved in the adaptation was approached – often this was the trial manager. If deemed appropriate, more than one individual involved in an adaptation was approached for interview. Individuals were emailed a copy of the patient information sheet (PIS) and consent form, with a reminder email one week after the initial email if no response, and a telephone call or email one week further if still no response. If the participant agreed to participate, a convenient time and date for the interview was scheduled.

Consent to participate in the semi-structured interviews was gained via a consent form, which was completed by the participant prior to the interview, and sent back to the researcher via email. The participant signed the consent form using an electronic (typed, or image of their signature inserted into the form) signature. The form was then countersigned by the researcher, and a copy of the completed consent form emailed or posted back to the participant.

##### Data collection

Semi-structured interviews were carried out with individuals based at the participating CTUs who were involved in implementing the adaptation. A semi-structured topic guide was used and covered topics including details of the adaptation, lessons learnt, challenges and benefits, and the potential impact of the adaptation on the efficiency of future trials. Interviews were carried out by RC (a male Research Associate with a BSc who worked in a CTU) and KS (a female Research Assistant with an MSc who worked in a CTU). Both interviewers had previous experience of qualitative interviewing. Repeat interviews were not carried out, and transcripts were not returned to the participant for comment or correction. There were no other individuals present at the interviews. Interviews lasted from 27 to 146 min.

All interviews were undertaken via Google Meet, with the audio from the interview recorded (with consent) using in-built functionality within the Google Meet platform and transcribed for in-depth analysis. As COVID-19 social distancing rules at the time meant that CTU staff were encouraged not to travel to the office, both the interviewers and the interviewees were at home when the interview was undertaken. Transcripts were anonymised prior to analysis.

Emphasis was placed on collecting detailed data from experienced participants. Participants did not provide feedback on the findings; however, non-participants did feedback on the themes within the workshop (WP4). Data saturation was not considered; rather, we looked to achieve ‘information power’, as conceived by Malterud et al., where the size of the study was determined by the amount of information the sample holds [17].

##### Relationships with participants

A relationship between three of the participants and KS (one participant) and RC (two participants) was already developed, due to the interviewees being based at the same CTU as the interviewers. There was no relationship already formed between any of the other participants and the interviewers, however, all interviewers were likely to have some knowledge of the interviewers and their goals.

##### Analysis

Data was analysed using thematic analysis, as described by Braun and Clarke [18]. NVivo software was used to manage the data. Analysis was undertaken by RC using the following steps: familiarisation, coding and identification of themes. The coding tree was split into two main themes – those related to individual adaptations, and those that cut across multiple adaptations. Within the former, there were codes for each general adaptation (e.g., remote consent), with sub-themes regarding discrete methods of undertaking these (e.g., telephone, or online), and then a third level of codes regarding the process of undertaking that implementation, the benefits, challenges, considerations for the future, and potential impact on efficiency.

### Literature review

A literature review was undertaken, focussing on the adaptations that were selected for detailed discussion in the semi-structured interviews. Broadly, the review consisted of three main search strategies for the three main adaptation types—recruitment, intervention delivery, and outcome assessment. Four recent systematic reviews relating to remote consent were located, and therefore, a further review of the literature was not undertaken [19–22]. For adaptations related to remote outcome assessment a search of the literature was undertaken, using MEDLINE and the search strategy outlined in Supplementary File 1. For remote intervention delivery, keywords were searched within Google Scholar, including combinations of keywords include “courier IMP”, “clinical trial”, “remote IMP delivery” and “remote delivery by CTU staff”. From both searches, the most relevant articles were selected following review by the corresponding author (RC). Searches were undertaken in March 2021.

### Workshop with CTU and patient representatives

The aim of the workshop was to seek the views of CTU personnel and patient representatives into the findings from the semi-structured interviews, including their views on the potential effect of the adaptations on efficiency.

#### Selection of workshop attendees

Potential workshop attendees were primarily identified from respondents to the survey that did not participate in the qualitative interviews. Additional attendees were identified from CTU webpages and from the project steering group, where the contact details of CTU staff who may be interested in trial adaptations (e.g., trial managers, directors) were collected. Public and patient involvement (PPI) representatives were approached who were already acting as PPI representatives for trials included in the survey. Additional PPI representations were identified from trials run by Sheffield CTU (the lead CTU which ran this study).

#### Workshop design

The workshop consisted of RC providing an overview of the findings for adaptations that were identified as potentially either directly or indirectly improving clinical trials. Those adaptations that were deemed to be pandemic specific, or where the impact was unknown, were not discussed within the workshop. After the findings were presented, the workshop attendees were split into small breakout groups, where the findings of the study were discussed, including their general reflections on the findings, challenges and benefits, and contexts in which the adaptation may or may not work in the future. Four study collaborators (RC, CC, CM and AoC) acted as facilitators. A group discussion was then held to feedback on the breakout group discussions.

### Results

#### Description of participants, studies, and adaptations.

Twenty-one of the 53 CTUs responded to the survey (response rate of 39.6%). Respondents described 40 studies that had made a total of 86 adaptations to recruitment, intervention delivery, or follow-up processes. From these, 14 studies were selected for interview, the characteristics of whom are described in Table 1.

**Table 1:** Interviewed participants and their characteristics

Study	Job title of interviewee	Gender of interviewee
Study A	Project Manager	Female
Study B	Senior Trial Manager	Female
Study C	Trial Manager	Female
Study D	Director	Female
Study E	Senior Trial Manager	Female
	Trial Manager	Female
Study F	Trial Set Up Coordinator	Female
Study G	Trial Manager	Female
Study H	Research Fellow	Female
Study I	Clinical Research Manager	Female
Study J	Trial Manager	Female
Study K	Trial Manager	Female
Study L	Trial Manager	Female
Study M	Trial Manager	Male
Study N	Research Assistant	Female

Eleven adaptations were identified from the selected studies (see Table 2), encompassing adaptations to the recruitment (four adaptations), intervention delivery (two adaptations) and outcome assessment processes (five adaptations)

**Table 2:** Adaptations selected for in-depth discussion within semi-structured interviews

**Group**	**Adaptation**	**Description**
**Recruitment adaptations**	Two-stage remote-first eligibility assessment	A two-stage eligibility assessment, where eligibility is assessed remotely prior to an in-person eligibility assessment
	Recruitment outside the NHS via a charity	The use of charities to identify and contact potential participants
	Remote consent (online or telephone)	The gaining of consent remotely, either via telephone or online
	Remote consent (postal)	Where consent for participation in the trial is obtained through the participant sending the consent form via the postal service
**Intervention delivery adaptations**	Couriering of the IMP to the participant	Where the study drug is sent to the participant, rather than having to attend a pharmacy
	Remote delivery of the intervention by CTU staff	Where CTU remotely deliver the trial intervention, instead of site-based NHS staff
**Outcome assessment adaptations**	Remote collection of PROMs, blood pressures and a measure of blood glucose	Telephone or postal collection of PROMs, and remote collection of biological measures—blood pressures and a measure of blood glucose
	Prioritisation of in-person assessments	Where the trial team contact the participant prior to a scheduled in-person visit to ascertain the safety or necessity of undertaking the assessment
	Prioritisation of in-person visits	Where the need to collect trial outcomes is reviewed for the entire trial
	Remote collection of spirometry and cough data	Where spirometry and cough data are automatically collected by a device and sent to the study team
	Collection of biological measures at another facility / use of routinely collected outcome measures	Instead of collecting the measure directly from the participant, another routine source is instead used

In the workshop, 11 CTU (three senior trial managers, three trial managers, two heads of research/CTU directors, two statistician and one data manager) and three patient representatives met to discuss the findings from the qualitative interviews.

### Literature review

#### Recruitment adaptations

Literature could not be located regarding two of the recruitment adaptations (a two-stage remote-first eligibility assessment and recruitment outside the NHS via a charity).

Remote consent procedures are generally well accepted across four recent systematic reviews [19–22]. Barriers identified include participant’s access to technology (particularly thought to be an issue in older adults) [19, 21, 22], and participants preferring traditional paper consent techniques, potentially due to issues around trust and data security [21, 22]. These reviews presented guidance for future studies, including the clinician or researcher being present to answer questions [20–22], seeking patient input into the consent materials [20], and using interactive features to aid comprehension [22]. Previous studies have found that ‘research champions’ are important to the recruitment and consent process [23, 24].

#### Intervention delivery adaptations

Evidence could not be located regarding either of the intervention delivery adaptations. Couriering the IMP to the participant has been discussed by several review articles since the start of the pandemic, however there are a lack of discussion of the challenges of benefits of doing so [25–27].

#### Outcome assessment adaptations

The prioritisation of in-person visits and assessments could not be located in the literature.

High response rates were identified for online data collection of PROMs when compared to email, telephone or mail follow-up across two studies [28, 29], and high response rates when tested alone [30, 31]. Online data collection was also deemed to be the most acceptable to participants across two studies [31, 32]. Reminders are important when undertaking online questionnaires [33]. Providing participants a choice of return methods (online or paper) resulted in a higher response rate than online only in one study [34].

The accuracy of remote data collection has been assessed, with computer assisted data collection reported to be as accurate as paper surveys [35, 36]. However, in two other studies, there were differences in responses to the questionnaires when comparing telephone vs mail, or paper to electronic versions [37, 38]. In one systematic review aiming to review modes of collection of subjective outcomes, the mode of administration (in person or remote) was significantly associated with bias, but not changes to precision [39].

The remote collection of blood pressures has been discussed as being acceptable for patients receiving clinical care (e.g. outside of a research setting), in populations such as pregnant or recently pregnant women, and individuals with heart failure [40–42]. However, there is a lack of evidence regarding acceptability within clinical trials and older adults.

### Impact of the adaptations

The potential impact of the adaptations on study efficiencies, including the potential challenges of implementation, and considerations for future studies, are presented in Supplementary Table 1.

Six adaptations (remote consent (postal), remote delivery of the intervention by CTU staff, prioritisation of in-person assessments, prioritisation of in-person visits, remote collection of spirometry and cough data and collection of biological measures at another facility / use of routinely collected outcome measures) were thought by the interviewees to be either inefficient, only applicable during the pandemic, or there was insufficient information collected in the interviews to assess the potential value in future trials. These were therefore not discussed in the workshop.

Five adaptations (two-stage remote-first eligibility assessment, recruitment outside the NHS via a charity, remote consent, couriering the IMP to the participant, and remote collection of PROMs, blood pressures and a measure of blood glucose) were deemed by the interviewees to have the potential to improve the efficiency of future trials and were discussed in the workshop (see Supplementary Table 1). Workshop attendees agreed with the findings from the qualitative study, and expanded on the themes identified from the semi-structured interviews, rather than challenging them.

Five themes relating to these potentially efficient adaptations, which were deemed by the authors to be the most pertinent to future studies, are summarised below.

#### Theme 1: Some adaptations may have a direct impact on trial efficiencies at NHS sites, but may only be applicable to certain circumstances.

Although interviewees from the 14 studies identified 11 adaptations they made during the pandemic, they perceived that only three of these had the potential to offer efficiencies. There were a lack of efficacy savings for CTUs, with any impact on efficacy being had at the NHS site level.

The first of these potentially efficient adaptations was a two-stage remote-first eligibility assessment, which may reduce trial costs by potentially saving trial sites time and resources in avoiding in-person visits for those who are not eligible for the trial.

It just makes it more efficient in terms of the participant’s time as well if they are not eligible and they’re not having to travel to a site, also in terms of expenses so you would normally pay you know, patient travel expenses that’s something you would save on. Study C

Interviewees discussed that, as this adaptation is particularly resource intensive for CTUs, it may only be relevant to smaller studies. Workshop attendees stated that this adaptation may only lead to efficiency in trials that involve a high number of ineligible participants being initially identified (e.g., recruitment via social media platforms).

Secondly, recruitment outside the NHS via a charity was perceived to potentially avoid the need for NHS staff input into recruitment and allowed more individuals to be approached in a shorter amount of time.

I think if you can access people [via charities] it’s a good way of reaching more people quickly. Study B

Both interviewees and workshop attendees felt that this adaptation may lead to bias (see theme 3), so may only be used as an adjunct to ‘traditional’ recruitment techniques.

Lastly, remote consent may make it easier for patients to take part in the trial, potentially increasing recruitment rates and reducing the recruitment phase of the trial – however there is insufficient evidence that this is the case.

I think it's quite efficient. For us, I think the more options that you have available [the better]. Study A

Access to technology is a particular issue for this adaptation (see theme 5), which may impact the scientific integrity of the trial.

#### Theme 2: Offering more options to trial participants may be more important than increasing efficiency

Allowing trial participants the flexibility to undertake trial procedures in their preferred manner was seen as important, and in some cases, was considered to be more important than directly improving the efficiency of the trial. Two of the 11 adaptations were thought to be unlikely to directly reduce the cost of future trials (i.e., they did not directly save the trial sites or CTU time) but benefitted trial participants through improving the flexibility by which trial procedures could be completed. These were: couriering of the IMP to the participant and remote collection of PROMs, blood pressures and a measure of blood glucose.

These adaptations may indirectly reduce trial costs by improving recruitment and retention rates, and thereby reducing the resources required for the trial. However, concerns were raised which may limit the use of these adaptations – the remote collection of outcome measures (blood pressure and PROMS) may affect the scientific validity of the trial (see theme 3); remote collection of blood glucose levels is clearly only relevant to specific populations.

Flexibility was not only important for participants but also for trial sites; increased flexibility could potentially make the trial look more feasible to potential research sites.

This is a huge selling point for us particularly because research and development (R&D) departments are quite reluctant to take on new studies at the moment, quite rightly they’re under a lot of pressure. And it’s a huge selling point for us to say we have this full flexibility and it’s fully remote if you want it to be. And it certainly is a benefit to the trial to have that. Study J

There are further recommendations regarding the use of these two adaptations which can be found in Supplementary Table 1.

#### Theme 3: Concern around the impact of the adaptations on the scientific validity of trials

All the adaptations were thought to have some impact on the scientific validity of the trial, which was a concern for workshop attendees. Adaptations to the recruitment process may impact on the ‘sampling frame’ of the trial, for example, by only recruiting those with access to technology (for remote consent adaptations), whilst couriering of the IMP to the participant may impact on the generalisability of trial results, as in real-world settings the IMP might not be delivered in this manner.

Adaptations to follow-up processes may skew the outcomes data – either when the adaptation completely replaces the ‘old’ way of collecting the data, or when undertaking an adaptation alongside the ‘traditional’ data collection procedure, potentially causing two distinct populations to be formed. This may occur when participants systematically undertake the outcome assessment procedure differently in one setting (e.g., at home), compared to the other (e.g., within the clinic setting). This may be particularly the case for the remote collection of blood pressures, where participants at home may select the ‘best’ reading to report, or may be more relaxed in their home environment, thus creating a different measure to those whose blood pressure in measured in clinic. The remote collection of PROMs may not have been validated for specific measures, potentially eliciting different responses to the questions to those individuals who complete the measure in clinic.

#### Theme 4: Limitations to the role of the CTU may be a barrier to implementing the adaptations

Limitations to the CTU’s role had the potential to affect each of the adaptations.

Many of the adaptations involved the transfer of trial procedures from the trial sites to the CTU—there was an impact on the resources required at the CTU. Some adaptations (e.g., couriering of the IMP to the participant’s home, and remote follow-up) required CTU staff to sometimes work outside of ‘normal’ working hours to fit in with the participant’s schedule.

There were examples of where the CTU were not best placed to fulfil the role of the trial sites. For example, the CTU may not have the clinical expertise available to collect clinical measures, and furthermore, CTIMPs may require a medically qualified individual to undertake certain trial procedures.

Maintaining a good relationship with trial participants was an important aspect of the adaptations. Interviewees expressed that the CTU were unlikely to have a pre-existing relationship with the participant, which may impact on the likelihood of the participant to take part in the trial or to provide outcome data.

Interviewees felt that maintaining a good relationship was especially important in the trials that involved participants with chronic conditions – e.g., trials involving participants living with motor neurone disease (MND), or parents of children with autism. In some cases, keeping close relationships with participants could outweigh the importance of efficiency.

I’d say, for this participant group, with an intervention that’s quite hands-on, and time-consuming, that local relationship, to me, seems more important than streamlining, or doing everything centrally where you’ve got total control over it. Study M

Workshop attendees stated that there may be limitations to the data that the CTU can collect. If the CTU does not have the necessary regulatory approvals to collect identifiable data, they may find it challenging to undertake activities that involve contacting the participant.

#### Theme 5: Limited access to—and negative impact of—technology

Technology was an important component of each of the adaptations, except for couriering of the IMP to the participant, for which technology had a limited impact. Access to technology was not only an issue for trial participants, but also for CTUs. Many adaptations were based on the participant utilising digital technologies; Interviewees were aware of the potential difficulties of participants utilising such technology; in two studies interviewees described how participants had dropped out of the trial due to issues around using technologies.

I think there might be a few that drop out, because the technology fear side of things. Study G

Many adaptations involved the participant undertaking tasks usually undertaken by the trial site or CTU, which put an extra burden on the participant. This, coupled with the reliance on technology, meant that training was required to try to support participants, either prior to them using the technology (i.e., training to prevent issues occurring), or in an ongoing manner in case of any issues (i.e., reactive support).

The CTU’s access to technology also impacted the adaptations that could be implemented. In a few cases, the exact method of implementing the adaptation was guided by the technology the CTU already had access to at the start of the pandemic. If they did not have the relevant software, then they did not make adaptations.

At the time, we did not have software which was capable of delivering e-consent, and we now have redcap, we didn't have at that time. Study A

The use of remote trial procedures could have a negative effect on the ‘quality’ of the trial procedure or data collected. Non-verbal signals may be missed during recruitment procedures, or important side effects may be missed when collecting outcome data.

I think that there are some participants that I would feel much better if I had them in a room in front of me and I can see their body language and I can see if they’ve understood what I’ve said and if they look like they feel a little bit unsure or you know you can tell it better face to face. You pick up on cues can’t you better so I guess that could be a drawback as well. Study J

## Discussion

### General findings

We undertook a survey of UK CTUs, with 21 CTUs (39.6%) providing information regarding adaptations that were made to their clinical trials. Of the 14 studies and 11 adaptations investigated, three adaptations were thought to have the potential to improve efficiency directly by reducing resources required at NHS trial sites: a two-stage remote-first eligibility assessment, recruitment outside the NHS via a charity, and remote consent. There was a lack of previously published evidence to support these adaptations, apart from remote consent, which is well supported by the literature. Other adaptations (remote collection of PROMs, blood pressures and a measure of blood glucose, and couriering the IMP to the participant) may benefit participants and indirectly benefit trials through increasing the appeal of participation in the trial. However, all the identified adaptations may only be applicable to certain trials and settings.

There are potential barriers to the implementation of these adaptations. Due to concerns around the effect of these adaptations on the scientific validity of trials (e.g., changes to the sampling frame for recruitment adaptations, and outcome assessment bias), the majority of adaptations were perceived to only be useful in future trials as an adjunct to more traditional methods. However, even using certain adaptations as an adjunct may cause bias, if there are systematic differences in the way an outcome is collected remotely, compared to in-person [37–39]. Additionally, CTUs may struggle to undertake these adaptations due to limited infrastructure (e.g., computer systems for online consent, and limited staff capacity to undertake centralised trial tasks, especially outside of usual working hours), and a lack of clinical expertise to collect clinical measures.

### Comparison to existing literature

The results of this study contradict evidence from one survey that found the majority of researchers (90%) felt that remote data collection processes made during the pandemic have the ability to improve the conduct of future studies [14], and a qualitative study of stakeholders involved in remote trials generally supporting the use of remote trial processes [43]. However, these studies have not focussed on the potential for these adaptations to improve the efficiency of future trials – in doing so, our study has found that although these adaptations may be generally acceptable to key stakeholders, there are barriers, and only a small number of adaptations may improve efficiencies in specific contexts.

We could not locate discussion of many of the identified adaptations in the literature, including a two-stage remote-first eligibility assessment, recruitment outside the NHS via a charity, and many of the adaptations for we found there was insufficient information to ascertain the effect on efficiencies (remote delivery of the intervention by CTU staff, delivery of trial intervention by an interventionist at any NHS Trust). However, this is not to say these adaptations are not already being used within clinical trials – many researchers may already be aware of many of these adaptations, but without detailed a description and evaluation of each adaptation, they may be challenging to implement.

Researchers should be aware of the wider implications of modifying clinical trials, especially when moving from in-person to remotely conducted procedures. The presence of clinical staff during recruitment activities promotes recruitment [20–22, 24], which may be limited when using remote recruitment procedures. Remote recruitment procedures may also result in lower recruitment rates, possibly due to participants preferring paper consent due to concerns around trust and data security [21, 22], which should be taken into account when planning the trial [44].

The online collection of PROMs was not identified as an adaptation that was widely used during the pandemic in this study, however, this adaptation is extensively represented in the literature, with higher response rates, accuracy and user acceptability when compared to other data collection techniques [28–32]. The speed at which adaptations needed to be made during the pandemic may have meant that there was not time to develop these complicated and onerous online collection systems.

We identified that a major barrier to implementing the identified adaptations were concerns around the effect on the scientific integrity of the trial. Other studies have also identified biases when different modalities are used to collect the same data [37–39], with one large systematic review identifying an impact on bias, but not precision [39].

### Strengths and limitations

A strength of this study is that all registered CTUs in the UK were surveyed to obtain details of studies that had made adaptations to continue during the pandemic at a time when the impact of the COVID-19 pandemic were still evident. The survey had a moderate response rate, with 21 of 53 CTUs (39.6%) reporting adaptations. Detailed feedback on the results of the study were sought from a workshop of CTU and patient representatives, where the challenges of implementing the adaptions were expanded upon, rather than challenged.

There are several limitations to this study. Firstly, the selection of 14 out of 40 studies may have resulted in novel or particularly effectual adaptations being missed. However, the studies were selected purposefully, ensuring variation in key characteristics. Secondly, only CTU representatives were interviewed, therefore representing a single perspective of the adaptations made, and excluding the views of trial sites and participants. Third, the contextual factors of undertaking research during the pandemic cannot be ignored – the motivation for trial participants, CTU staff, and other stakeholders (regulatory bodies, sponsors) to enable research to continue during the pandemic may have been a major enabling factor that allowed the adaptations to function. Such motivation may be unachievable outside of the pandemic. Additionally, a relationship was already formed between the interviewees and interviewers for three participants of the qualitative study – however this did not lead to any discernible bias on the data collected. Lastly, the survey sent to CTUs was not rigorously tested prior to use and may therefore have lacked content and/or face validity. However, the effect of this is likely to be minimal, as the survey collected only basic descriptive data about the adaptations undertaken. There was no indication that any of the questions were misinterpreted by respondents.

### Implications & future research

#### Implications

In this study, we have identified adaptations that may be used in specific trials or populations, which may lead to benefits for the NHS sites and/or trial participants. With the information gained from this study, clinical trialists can learn about adaptations that can be implemented in specific circumstances and potentially increase trial efficiency. We have identified important factors for researchers to consider when implementing an adaptation, including the importance of the relationship with the participant, the suitability of CTUs to undertake adaptations, and the importance of allowing the participant flexibility.

However, the findings from this study may be challenging to implement. Clinical trials have previously been slow to implement new technologies, possibly due to concerns around confidentiality, poor infrastructure, and data accuracy [45]. These concerns are likely to prevail, especially in relation to scientific integrity, which may prevent the use of the adaptations not only as stand-alone adaptations, but also as adjuncts alongside the traditional method of undertaking the trial procedure, therefore limiting the flexibility that participants can be provided.

### Future research

We have identified adaptations that potentially improve the efficiency of RCTs, however, the conclusions are based on the perceptions and experiences of CTU staff. It is also important to measure the impact of these adaptations because as there is a lack of evidence to support them in the literature. Studies within a trial (SWATs) could be used to quantitatively evaluate the effect of the adaptations on key trial variables [46]. The experience of trial teams of implementing these adaptations could also be reported and shared within journal articles. However, although it would be beneficial to undertake evaluations of the adaptations identified in this study, the use of these adaptations by researchers may promote a perceived utility, enabling their use more widely.

## Conclusions

Of the 11 adaptations selected for in-depth assessment from those that were made to trials by UK CTUs during the pandemic, there were a lack of adaptations that were perceived to directly impact on trial efficiencies at CTUs. Three adaptations (two-stage remote-first eligibility assessment, recruitment outside the NHS via a charity, and remote consent) may directly improve the efficiency of trials at NHS sites, by reducing the resources required, however there is a lack of published evidence to support the use of the former two adaptations. Other adaptations may indirectly improve the efficiency of trials by improve the flexibility by which participants can undertake trial procedures, therefore making the trial more appealing for participants. All the adaptations were only thought to be applicable to specific circumstances, and all had their limitations, the most significant of which were the effect of the adaptations on the scientific validity of the trial. Online data collection, which is widely reported in the literature as being an accurate and acceptable data collection technique, was not represented in our sample, possibly because CTUs had limited time and resources to adapt trials. Future research should focus on the effect of the adaptations on key trial variables, including recruitment and retention rates, within SWATs.

## Supplementary Information

**Additional  file 1.** Outcome assessment search strategy.

**Additional  file 2. Supplementary Table 1:** Efficiency of the adaptations, challenges and benefits, and consideration**s**

## Data Availability

The datasets generated and/or analysed during the current study are not publicly available so that confidentiality can be maintained but are available from the corresponding author on reasonable request.
