# Supplementary material for: Learning from COVID-19 related trial adaptations to inform efficient trial design—a sequential mixed methods study
Source: BMC Med Res Methodol. 2022 Apr 29;22:128. doi: 10.1186/s12874-022-01609-6 (PMC9051017; doi:10.1186/s12874-022-01609-6)
Supplement: Supplementary file 1 — Additional file 1. Outcome assessment search strategy. [file 12874_2022_1609_MOESM1_ESM.docx]

OUTCOME ASSESSMENT SEARCH STRATEGY

1. ((((outcome* adj assessment*).tw. or follow*.mp.) adj up*.tw.) or follow-up*.tw. or assessment*.tw. or data.mp.) adj collect*.tw.
2. (telemedicine or video or multimedia or telecommunication* or computer* or online or remote* or internet or web or telephone or phone).tw.
3. (Clinical Trial or Randomized Controlled Trial).hw.
4. 1 and 2 and 3
