# Supplementary material for: Learning from COVID-19 related trial adaptations to inform efficient trial design—a sequential mixed methods study
Source: BMC Med Res Methodol. 2022 Apr 29;22:128. doi: 10.1186/s12874-022-01609-6 (PMC9051017; doi:10.1186/s12874-022-01609-6)
Supplement: Supplementary file 2 — Additional file 2. Supplementary Table 1: Efficiency of the adaptations, challenges and benefits, and considerations [file 12874_2022_1609_MOESM2_ESM.docx]

| **Supplementary Table 1: Efficiency of the adaptations, challenges and benefits, and considerations** | | | | | | |
| --- | --- | --- | --- | --- | --- | --- |
|  | | **Adaptation** | **Impact on efficiency** | **Studies/populations in which adaptation may be efficient** | **Challenges and benefits** | **Guidance or considerations** |
| Recruitment | | Two-stage remote-first eligibility assessment | Yes – direct impact on NHS sites | - Smaller studies - Studies not involving sensitive topics/questions - Studies requiring the participant to make a decision regarding their involvement in the trial prior to a fixed event (e.g., surgery). - Studies where eligibility assessments can be undertaken remotely by CTU staff. - Low risk studies. - Studies where a high proportion of participants can be screened out prior to an in-person visit (e.g., studies involving recruitment via social media). | **Challenges**   - Discussion of sensitive topics or questions over telephone may be challenging. - Reading participant’s expressions and body language is important and may be missed if undertaken remotely. - May be more challenging to describe recruitment procedure due to increased complexity. - Eligibility and baseline data no longer collected directly prior to randomisation. - If undertaken by CTU staff, clinical staff may have less ownership over the consent process. - May be problematic for CTUs to receive identifiable data if the participant is not self-referring. - Certain measures may not be validated for use outside the in-person clinic setting.   **Benefits**   - Ability to screen out participants early may save time. - Centralisation of eligibility process may allow faster completion. - May allow participant more time to consider participation in trial. - If undertaken by a CTU, may allow clinical staff more time to discuss the trial at a later appointment. | - Some investigations may need to be undertaken in person after the remote eligibility assessment (e.g., pregnancy test). - The eligibility process may be able to be undertaken quicker by a CTU, but overall, the process may take longer due to multiple steps. - May be unsuitable for studies that require a qualified medical professional to confirm eligibility. - Unlikely to be resource saving unless participants are screened out prior to an in-person eligibility assessment. |
|  |  | Recruitment outside the NHS via a charity | Yes – direct impact on NHS sites | - May be best used as an adjunct to recruitment within the NHS, rather than by itself, due to a potential impact on the sampling frame. - Low risk studies not requiring medical input into participant recruitment. - Studies for which there is a relevant condition specific charity which is suitability large. - Studies where potential sampling biases have a limited effect on the external validity of the trial. | **Challenges**   - Issues with participant sampling – either the charity not sampling correctly, self-selection bias, or inability to access population of interest. - Relationship between the participant and researcher/clinician is important – remotely conducting recruitment via a charity may impede this. - Low response rate (20% in one study) if emails are used. - The charity may not have the expertise or resources to conduct research processes.   **Benefits**   - Many participants can be contacted at once, potentially quicker than can be achieved by individually contacting participants within NHS Trusts. | - A range of recruitment techniques (involving both NHS and non-NHS routes) may be preferable. - Reminders required to prompt participants to complete recruitment steps. - Recruitment could be undertaken by CTU, unless study is high risk of a CTIMP, in which case a clinically qualified person may be required to confirm eligibility. - Charities may require training in recruitment processes, requiring time and input from CTUs and the charities. - Charities may not have the necessary information to be unable to identify those individuals who are too vulnerable to participate in the trial. |
|  |  | Remote consent | Yes – direct impact on NHS sites | - Studies where a close relationship between the researcher and participant is not critical. - Has the potential to increase efficiency by improving recruitment rates. | **Challenges**   - May impact on the participant-researcher relationship, if consent is undertaken remotely and/or by a member of CTU staff. - Risk of a shift in the sampling frame of the study if consent is obtained using a technology/platform that some potential participants are unlikely to have access to. - Digital literacy is a concern. - Consent may be more challenging to undertake remotely. - Not always possible to know if participants have been pressured by family or others when consent is not in person. - Sensitive conversations may be difficult to have remotely.   **Benefits**   - May enable participants more time to consider the trial. - Trial sites do not have to use limited clinic space to facilitate in-person consent. - Allows the participant flexibility. - May allow family members/friends to be present during conversation. | - Do not assume that the REC will not support a method of consent that may not be the ‘safest’ or most secure. - Remote consent (i.e., consent via telephone or video calls) may be easier to implement compared to electronic consent, for both participants and CTU, due to limited access to this technology. - Clear guidance to sites is important. - Sites with more motivated investigators may be more successful at gaining remote consent – more support may be required for other sites. - Reminders may be required to obtain responses from participants. - It may take significant resources for CTUs to develop remote consent procedures. - Multiple options or mediums of gaining informed consent may be required if there is a risk that using only one technique may bias the sample. - Some participants may benefit from in person informed consent – flexibility is key. - It may be necessary to maintain an audit trail of the consent conversations that are had if the participant isn’t able to physically sign the consent form. - If possible, avoid the need for participants to type a URL into a browser – this may result in participants making data errors and becoming disengaged from the recruitment process. |
| Intervention delivery | | Remote delivery of the intervention by CTU staff | No. Pandemic specific. |  | **Challenges**   - The scientific integrity of the trial may be impacted by the fact that the intervention is not being tested in the ‘real world’. - Range of facilitators reduced, meaning each facilitator may have an increase influence over delivery of the intervention.   **Benefits**   - A smaller, centralised team allows more controlled facilitation of the intervention - Direct feedback between participants and CTU staff |  |
|  |  | Delivery of the trial intervention by any interventionists at any NHS Trust | Unknown | - Studies with a HEI sponsor - Studies involving interventions that can be carried out remotely, where there are numerous trained individuals across the UK | **Challenges**   - Seeking excess treatment costs, transfer of data between Trusts, and agreement of whom takes responsibilities for the participant’s clinical care may be challenging.   **Benefits**   - May increase the pool of potential participants, therefore increasing recruitment throughput. | - Avoid including PIs who are not engaged in trial as receivers of external referrals. - Allows therapist absence at one site to be covered by therapists from other sites. |
|  |  | Couriering the IMP to the participant’s home | Yes – benefits participants | - IMPs not requiring strict temperature regulation - Studies that can incorporate costs for IMP couriering into their grant. | **Challenges**   - Significant resources required at site or the CTU to track and organise the courier, including outside of normal office hours. - Significant resources may also be required to review SOPs and formulate courier processes. - Expensive. - Logistical issues, including the requirement for wet ink signatures, and pharmacies closing before the courier attends. - May result in poor external validity, if, in the ‘real-world’, the drug would not be couriered to the participant.   **Benefits**   - Couriering the IMP may make the trial more desirable and increase participant recruitment and retention. | - Return of the IMP important to consider. - Confirmation that the participant has received the IMP may be required – either by directly contacting the participant or receiving notifications from the courier. - Ensure packaging is correct and the site pharmacy approve it. - Adherence data may be difficult to collect and be reliant on trusting the participant to provide reliable data. - Sites may automatically defer to using a courier and may need reminding that the participant can attend in-person. - Sites may need time to update their SOPs if they have not couriered medications in the past, which the CTU may need to review. - Between arm differences in how the drug is couriered may result in bias. |
| Follow-up | Remote collection of PROMs | Telephone & postal | Yes – benefits participants | - Studies may consider using this adaptation as a back-up for remote patients or those that cannot attend the study site | **Challenges**   - Risk of missing side effects when participant cannot be seen in person. - Data may be missing if limited guidance or input is provided to the participant when completing measures. - May be difficult to ask sensitive conversations remotely.   **Benefits**   - Allows trial participants increased flexibility in how trial procedures are undertaken, and therefore may improve recruitment rates through making the trial more appealing. - Telephone data collection may lead to particularly good compliance and low missing data but may be onerous for long questionnaires. - Postal data collection may require high levels of CTU input – including dealing with missing data and administering reminders to participants. | - Training may be required if CTU staff are to deliver PROMs. - Certain instruments may not be validated for use outside the in-person clinical setting. - Maintaining blinding was challenging in one trial. In order resolve this, each site has a blinded and non-blinded research assistant. - Participants may not pick up the telephone for outcome collection if being called from an unknown number. - As the clinical team may not be directly involved in the follow up, criteria may be required for stopping the IMP, e.g., in the case of high depression levels. - Telephone follow-ups may need to be split into multiple sessions if many measures are being collected and may require out of hours working at the CTU. - Postal data collection may require follow-up windows to be extended. - Repetitive question formats should be avoided over the telephone. Questions should be kept as simple as possible via all mediums. - Inform participant prior to the call the nature of the conversations to assist them in dealing with sensitive questions. - Trial sites can be involved in prompting participant for missing data or checking potentially erroneous or clinically concerning data. - There may be generational differences in the acceptability of different data collection techniques – the younger generation may not want to use the telephone, and may prefer text messages; older generations may prefer telephone. |
|  |  | Video | Unknown |  |  | - Used in a study involving populations with chronic conditions that reduce ability to communicate via other methods. |
|  | Remote collection of biological measures | Blood pressures collected remotely | Yes – benefits participants | - Studies where taking blood pressures remotely would avoid the need for participants to attend an appointment. - Studies where participants can measure their own blood pressure. | See also “remote delivery of PROMs”  **Challenges**   - Participants may provide erroneous values – e.g., supply their lowest blood pressure readings. - Potential for loss of data if readings are not automatically uploaded. - Concerns around calibration and quality of devices – good quality devices may be very expensive. - May work against inclusivity – e.g., those with chaotic lifestyles | - Unlikely to be more efficient, but more flexible for participants. - Compliance may depend on patient group or individual patient’s motivations, e.g., patients who are less engaged in their therapy may be less likely to provide accurate data. - Participants may want to see a clinician. - Participant’s readings may better reflect their ‘actual’ blood pressure levels when measured in the home environment. |
|  |  | Spirometry & cough data collected remotely | Unknown | - Studies collecting biological measures, where technology assists to collect the outcome remotely and automatically (e.g., spirometry data) - Studies that have the budget to invest in such technologies | **Challenges**   - Cost implications   **Benefits**   - Remote collection of spirometry data, and automated upload to the trial database, allowed for additional secondary outcomes to be collected. | - Unknown acceptability from participant’s point of view. |
|  |  | Remotely collected blood glucose measure (Hb1Ac) | Yes – benefits participants | - Studies where taking Hb1Ac remotely would avoid the need for participants to attend an appointment. - Studies where participants can measure their own blood glucose levels. | **Challenges**   - Extensive resources required at CTU to administer and send packs. - Potential poor response rate. - Participants accessing a post box to return the kit may be the most challenging part for more ill or vulnerable participants.   **Benefits**  See “remote delivery of PROMs” | - Unknown acceptability from participant’s point of view. - Need to ensure process isn’t too burdensome for participants. |
|  | Other | Collection of outcomes from a routine source | Unknown |  |  | - Cheaper and involves less travel for participants. |
|  |  | Prioritisation of trial outcomes or in-person visits | No. Pandemic specific |  |  |  |

CTU: Clinical Trials Unit; CTIMP: Clinical Trial of an Investigational Medicinal Product; HEI: Higher Education Institution; IMP: Investigational Medicinal Product; NHS: National Health Service; PI: Principal Investigator; PROM: Patient Reported Outcome Measure; REC: Research Ethics Committee; SOP: Standard Operating Procedure.
